# Development and Characterization of a Novel Non-Lytic Cancer Immunotherapy Using a Recombinant Arenavirus Vector Platform

**DOI:** 10.3389/fonc.2021.732166

**Published:** 2021-10-14

**Authors:** Henning Lauterbach, Sarah Schmidt, Kia Katchar, Xiaoping Qing, Corinne Iacobucci, Andy Hwang, Katia Schlienger, Ursula Berka, Josipa Raguz, Sarah Ahmadi-Erber, Timo Schippers, Felix Stemeseder, Daniel D. Pinschewer, Igor Matushansky, Klaus K. Orlinger

**Affiliations:** ^1^Hookipa Pharma Inc., New York, NY, United States; ^2^Department of Biomedicine - Haus Petersplatz, Division of Experimental Virology, University of Basel, Basel, Switzerland

**Keywords:** arenavirus, LCMV (lymphocytic choriomeningitis virus), HPV – human papillomavirus, cancer immunotherapy, HNSCC (head and neck squamous cell carcinoma), CD8+ T cells, Pichinde virus (PICV), active immunization

## Abstract

Engineered viral vectors represent a promising strategy to trigger antigen-specific antitumor T cell responses. Arenaviruses have been widely studied because of their ability to elicit potent and protective T cell responses. Here, we provide an overview of a novel intravenously administered, replication-competent, non-lytic arenavirus-based vector technology that delivers tumor antigens to induce antigen-specific anti-cancer T cell responses. Preclinical studies in mice and cell culture experiments with human peripheral blood mononuclear cells demonstrate that arenavirus vectors preferentially infect antigen-presenting cells. This, in conjunction with a non-lytic functional activation of the infected antigen-presenting cells, leads to a robust antigen-specific CD8^+^ T cell response. T cell migration to, and infiltration of, the tumor microenvironment has been demonstrated in various preclinical tumor models with vectors encoding self- and non–self-antigens. The available data also suggest that arenavirus–based vector therapy can induce immunological memory protecting from tumor rechallenge. Based on promising preclinical data, a phase 1/2 clinical trial was initiated and is currently ongoing to test the activity and safety of arenavirus vectors, HB-201 and HB-202, created using lymphocytic choriomeningitis virus and Pichinde virus, respectively. Both vectors have been engineered to deliver non-oncogenic versions of the human papilloma virus 16 (HPV16) antigens E7 and E6 and will be injected intravenously with or without an initial intratumoral dose. This dose escalation/expansion study is being conducted in patients with recurrent or metastatic HPV16+ cancers. Promising preliminary data from this ongoing clinical study have been reported. Immunogenicity data from several patients demonstrate that a single injection of HB-201 or HB-202 monotherapy is highly immunogenic, as evidenced by an increase in inflammatory cytokines/chemokines and the expansion of antigen-specific CD8^+^ T cell responses. This response can be further enhanced by alternating injections of HB-202 and HB-201, which has resulted in frequencies of circulating HPV16 E7/E6-specific CD8^+^ T cells of up to 40% of the total CD8^+^ T cell compartment in peripheral blood in analyses to date. Treatment with intravenous administration also resulted in a disease control rate of 73% among 11 evaluable patients with head and neck cancer dosed every three weeks, including 2 patients with a partial response.

## Introduction

Immunization strategies that induce clinically effective cytotoxic T cell responses against tumors have the potential to address critical unmet needs in the treatment of cancer. Researchers are continuing to explore novel strategies to harness and optimize viruses as cancer immunotherapeutics (1, 2). Next-generation viral vectors have been created to induce more potent tumor antigen-specific T cell responses and to overcome the challenges of an immunosuppressive tumor microenvironment (TME) (1, 2). Engineered arenavirus-based vectors are a promising new strategy that is currently being studied preclinically and in the clinical setting (3). Preclinical data have demonstrated that these vectors can result in a robust activation of antigen-specific T cells and release of pro-inflammatory cytokines that can provide antitumor effects and overcome immunosuppression in the TME (2, 4, 5).

Mammalian arenaviruses are bi-segmented ambisense single-stranded RNA viruses that are divided into two groups: Old World and New World (6). This classification was originally based on geographic distribution and serological relatedness, and later confirmed by phylogenetic analysis of genome sequences (6). Arenaviruses, including lymphocytic choriomeningitis virus (LCMV) and Pichinde virus (PICV), have been studied for many decades as model systems that induce extremely potent and long-lived virus-specific T cell immunity (7–12). While LCMV is prevalent in wild mouse colonies worldwide, its human seroprevalence (as measured by antibodies against LCMV) is estimated to be approximately 2% to 5% in most countries (13, 14). In immunocompetent individuals, LCMV infections are generally asymptomatic or manifest as self-limiting flu-like symptoms (13, 15, 16). Similarly, PICV infection of humans, albeit asymptomatic, has been documented in the context of laboratory exposure, with no known associated human disease (12, 17–19).

Accordingly, the percentage of the human population with preexisting anti-vector immunity due to prior exposure to the wild-type virus is negligible. This contrasts with other commonly vectorized viruses (eg, adenoviruses) for which preexisting immunity to certain serotypes used in vaccines can limit immunogenicity and clinical responses (20, 21).

While arenavirus infections induce strong humoral and cell-mediated immune responses, it has been known for decades from preclinical models that neutralizing antibody (nAb) to arenavirus infections commonly arise very late in the infection cycle and reach only low titers (22). The mechanism underlying this nAb evasion derives from the presence of multiple glycans on the envelope proteins of many arenaviruses (22, 23). These glycans obstruct antibody access to neutralizing epitopes, thereby hindering antibody neutralization of infectious virions (22, 23). In the absence of nAbs, initiation of a strong immune response toward wild-type LCMV and PICV can develop by their direct infection of antigen-presenting cells (APCs). One mechanism by which LCMV infects APCs is *via* binding to its cellular receptor, α-dystroglycan (24, 25). Normally, α-dystroglycan mediates interactions between the extracellular matrix and the intracellular cytoskeleton and is expressed on numerous cell types, including APCs (25). The cellular receptor used by PICV for infection is currently unknown, but PICV has been shown to infect and activate human macrophages (26). LCMV infection activates APCs and triggers a robust CD8^+^ T cell response to viral antigens (27, 28). Notably, the infection cycle of arenaviruses is non-lytic and does not kill the host cell (6).

Cytotoxic T lymphocytes (CTLs) are critical to immunity against viruses and other intracellular pathogens and are essential in eradicating cancer cells (29). For numerous solid tumors, the presence of high levels of tumor-infiltrating lymphocytes (TILs) within the TME has been shown to predict more favorable outcomes and higher response rates to immunotherapies (30–37). Therefore, a key goal of immunotherapy is to convert non-inflamed tumors, so called “cold” tumors, into inflamed “hot” tumors, which are characterized by high levels of TILs and other markers of immune activation (38). Mouse tumor models have shown that therapeutic treatment can promote this “cold” to “hot” transition by activating antigen-specific CD8^+^ T cells that travel to and infiltrate the tumor and release pro-inflammatory cytokines in the TME (4, 39).

Viral vectors engineered from LCMV and PICV, described in this review, were attenuated by means of artificial genome organization. The arenavirus genome consists of two negative-stranded RNA segments (S- and L-segments) encoding two viral genes each, with untranslated regions and an intergenic region flanking the open reading frames on each segment. Integration of genes of interest in replicating arenavirus vectors has been achieved by redistributing the viral genes from two to three genome segments with duplicated S-segments, each encoding one viral gene and one antigen gene of interest (40). To prohibit formation of a nonattenuated wild-type virus by intersegmental recombination of the duplicated S segments, artificial positioning of the viral genes on the S segments was applied. This prevents reformation of a wild-type S segment by generating an inactive recombination product devoid of a viral promoter (5). These genetic modifications should not affect tropism of the resulting vector because proteins influencing tropism such as the viral surface protein were left unchanged. The safety of these engineered arenaviral vectors has been extensively documented in preclinical studies, and an encouraging safety profile is emerging from clinical trials (4, 5, 39, 41).

Taken together, these properties make arenaviruses a particularly attractive platform for use as cancer therapies. In this review, we describe systemically administered, replication-competent arenaviral vectors, which induce high levels of tumor-specific CD8^+^ T cell responses (4, 5, 39). Data from cancer models using arenavirus vectors expressing self- or non–self-antigens and results from vectors in clinical development for the treatment of advanced or metastatic human papilloma virus positive (HPV+) cancers will be summarized. More details on the results and methods used in these experiments have been previously published or presented (4, 5, 39, 41, 42).

## Differentiated Mechanism of Action and Immunogenicity of Arenavirus Vectors

The preclinical research to date on this platform, summarized as follows, has focused on the evaluation of immunogenicity, safety, and antitumor activity. The goals of these experiments were to investigate and describe the virological and immunological mechanisms underlying the exceptionally potent CD8^+^ T cell activation and tumor control mediated by these vectors. Key mechanistic steps, including vector spread to lymphoid organs, APC infection and activation, robust activation of polyfunctional antigen-specific CTLs, and migration of activated CTLs to the tumor, are summarized.

This platform of replication-competent arenavirus vectors has been engineered to express a broad range of antigens relevant to cancer, including non-self oncoviral antigens (eg, E7 and E6 from HPV), but also cancer self-antigens (e.g., cancer testis, differentiation and overexpressed antigens), and neoantigens. Preclinical research has been conducted to profile several engineered LCMV- and PICV-based vectors, including experiments in mouse models of HPV+ cancer, melanoma, mastocytoma, and thymoma.

### Systemic Administration of Replication Competent Arenavirus Vectors

**Figure 1:**
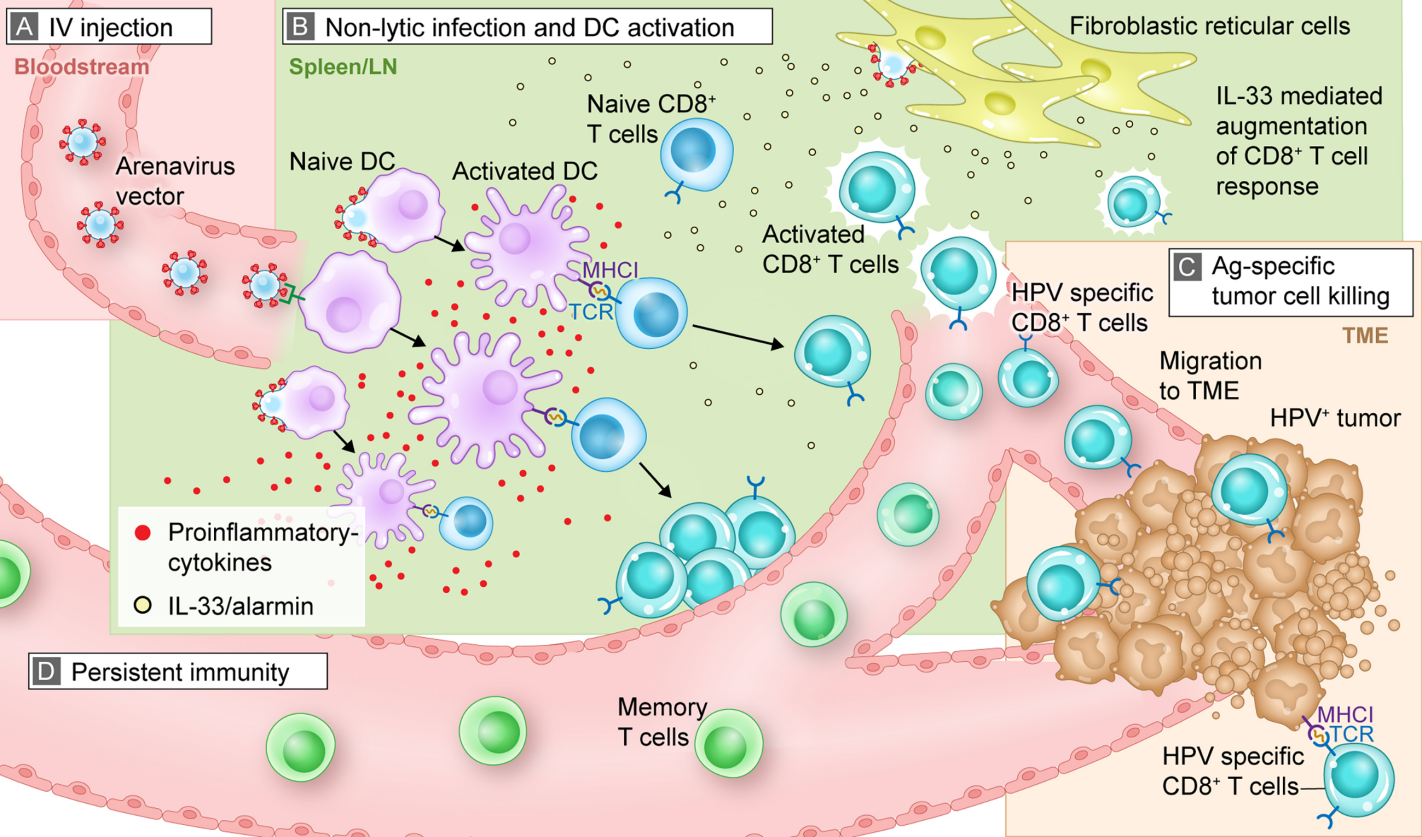
Mechanistic summary of how arenavirus vectors are hypothesized to exert their effects *in vivo* to trigger an antitumor, antigen-specific immune response. This graphic representation uses HB-201 and an HPV+ tumor as an example for illustrative purposes. **(A)** After IV injection, HB-201 rapidly enters the bloodstream, where viral vectors travel to lymphoid tissue; **(B)** once in the lymphoid organs, HB-201 vectors infect and activate APCs such as DCs. Antigen-specific CD8^+^ T cells are then activated and proliferate in the presence of key inflammatory cytokines and the alarmin cytokine, IL-33, which is secreted by infected fibroblastic reticular cells; **(C)** polyfunctional T cells migrate to the TME, where they interact with and kill cancer cells expressing HPV E7 and E6 antigen epitopes *via* MHCI; **(D)** development of memory T cells provides persistent immunity that can protect from tumor recurrence. Ag, antigen; APC, antigen presenting cell; DC, dendritic cell; HPV, human papilloma virus; IL, interleukin; IV, intravenous; MHCI, major histocompatibility complex class I; TCR, T cell receptor; TME, tumor microenvironment.

Recombinant arenavirus vectors can be delivered by different routes of administration, including systemic (intravenous [IV]) or local (intratumoral) administration which have been used in the preclinical studies described in this review. Clinical studies are expected to use primarily IV dosing, as this mode of administration can be used in all patients and is minimally invasive. This contrasts with intratumoral injection, which requires access to tumors that may not be possible for a portion of patients. Further, IV injection allows for the efficient distribution of arenavirus vectors through the bloodstream to reach a large pool of APCs (**Figure 1A**). After IV administration, the vector is found in multiple organs including liver and spleen, and is rapidly cleared without inducing tissue damage or protracted viremia (5).

Various strategies have been employed to optimize antigen-specific immune responses induced by technologies such as RNA-, DNA-, peptide- or protein-based immunizations, which commonly utilize intramuscular, intradermal, or subcutaneous routes of administration. However, after local injection, the antigen is primarily expressed and/or presented by cells of local solid tissue rather than APCs (43–45). Local migratory DC subsets are then required to transport the antigen to lymph nodes, where it is presented to CD8^+^ T cells (46, 47). Accordingly, the uptake of apoptotic or necrotic tissue fragments and their processing and presentation on major histocompatibility complex (MHC) class I molecules (so-called cross-presentation) appear to be the main mechanism for generating CTLs following this type of vaccination (43, 48, 49). While tumor-specific immune responses can be induced using various immunization modalities, mimicking the natural course of viral infection, including delivering antigens by activating professional APCs and instructing them to produce themselves the virally encoded antigens for presentation (so-called direct presentation), promises to yield T cell responses superior to modalities that do not activate the immune response by this mechanism (50, 51). By directly infecting APCs, arenavirus vectors can mimic this natural course of infection and immune activation systemically, which offers a potential advantage over other types of therapeutic immunizations.

As previously noted, prior research has shown that glycans on the arenaviral envelope hinder antibody-mediated neutralization of wild-type arenaviruses (**Figure 1A**) (22, 23). This observation, combined with low seroprevalence in humans, suggests that there is low risk of preexisting nAbs from a prior infection or due to vector administration. To assess this hypothesis, preclinical and clinical studies were conducted to determine if infection with arenavirus vectors would induce vector-neutralizing antibody responses. In a placebo-controlled phase 1 safety and immunogenicity study, 42 healthy volunteers were vaccinated with three administrations of non-replicating LCMV-based vectors expressing the cytomegalovirus antigens gB and pp65. LCMV-vector nAbs were detected in only 1 of 42 patients at one time point (month 4). At 12-month follow-up, nAbs were no longer detected in this patient (52). These results were supported by data from a mouse cancer model that showed undetectable levels of vector nAbs induced by replication-competent LCMV and PICV vectors after the second dose (4, 5). The development of nAbs can be a limitation to vaccine efficacy because, in part, nAbs block the ability to achieve greater immunity after multiple doses (53). In contrast, the lack of nAbs against arenavirus-based vectors allowed repeat injections to enhance T cell responses against the encoded antigens. LCMV and PICV vector nAb levels are also being monitored as part of the ongoing phase 1/2 trial in patients with HPV16+ cancers (3).

### Drainage to Lymphoid Tissue and Activation of an Immune Response

Once in the bloodstream, the arenavirus vectors quickly reach the lymphatic system (spleen or lymph nodes), where they infect and activate APCs and initiate a cascade of immune activation (**Figure 1B**). As previously described, LCMV preferentially infects APCs, including dendritic cells (DCs), by mechanisms which include virus binding to its cellular receptor, α-dystroglycan (24, 25). Preclinical studies confirmed that replication-competent LCMV vectors preferentially infect monocytes, macrophages, and DCs over T and B cells (5, 54).

DCs infected with LCMV vectors exhibit an activated phenotype, as demonstrated by higher levels of cell-surface CD86 (a marker of DC activation), compared with non-infected DCs (54). These findings support the interpretation that LCMV vectors can directly infect and activate APCs. Infection with arenavirus vectors or wild-type arenaviruses is non-lytic (i.e., replication and generation of progeny viruses do not kill the infected cell). This non-lytic infection may prolong the time for antigen presentation and activation of a robust immune response.

Since arenavirus vectors directly infect APCs, they do not need to infect tumor cells to be efficacious. In contrast, oncolytic viral cancer therapies developed and evaluated clinically to date require direct cancer cell infection and lysis to be effective (55). In general, this feature requires the vectors to either be administered locally (intratumorally) or to localize to the site of the tumor, which can be deeply embedded in the tissue, rendering it an inefficient process (56). While these types of vectors may be valuable in the treatment of patients with localized disease, they may be less effective in patients with advanced disease, in whom it can be difficult to identify and access all metastases, including micrometastases. Arenavirus vectors, when administered intratumorally in preclinical models have been shown to infect tumor and fibroblastic stromal cells (FSCs) in tumors and thereby induce profound activation and reprogramming of the TME, such as release of certain cytokines that sustain TIL functionality and reduce TIL exhaustion, which leads to sustained control of tumor growth. Importantly, beyond local effects, the intratumorally administered arenavirus vectors induce systemic antitumor immunity, which have been shown to also constrain tumor growth in peripheral tissues (57). When administered systemically, arenavirus vectors trigger a systemic tumor antigen-specific immune response without having to localize to the tumor. In this way, the arenavirus vector–induced T cells migrate to the tumor, inflame the TME, and eradicate the tumor cells (4, 5, 39).

### Induction of Antigen-Specific Immune Responses

After infection with arenavirus vectors, APCs mature and synthesize the vector-encoded antigens. Together with appropriate costimulatory signals (eg, CD80/CD86 and secreted cytokines), the processed antigens are presented to CD8^+^ T cells *via* MHC class I molecules in a process referred to as direct presentation (**Figure 1B**). This process contrasts with cross-presentation, in which exogenous antigens from other infected cells are phagocytosed by DCs and then presented to T cells. The infection of professional APCs, including monocytes and DCs, all capable of direct presentation, together with potent costimulation is an advantage of arenavirus vectors over other antigen delivery systems. Importantly, the use of arenavirus vectors is also not limited to specific patient MHC haplotypes. For example, short peptide-based vaccines that directly bind to MHC molecules require patients who express a matching human leukocyte antigen (58). The same applies to T cell receptor-transgenic “TCR” approaches (59). In contrast, arenavirus vectors can deliver full-length proteins, thereby overcoming this potential limitation.

Infection and activation of DCs in the presence of key pro-inflammatory cytokines lead to the activation of antigen-specific CD8^+^ T cells. The experience from several decades of research in mouse models has shown that arenaviruses are extremely efficient at this task, and this feature is maintained by replication-competent arenavirus vectors (4, 5). The naturally potent CD8^+^ T cell induction by arenaviruses can be extended to antigens encoded in the replication-competent arenavirus vectors, which are also highly immunogenic. Injection of replication-competent arenavirus vectors thereby results in high frequencies of antigen-specific CD8^+^ T cells directed against the vector-delivered antigen. A broad range of self- and non–self-antigens have been evaluated in preclinical models. For example, the antigen-specific immunogenicity of a replication-competent LCMV vector that expresses HPV16 E7 and E6 genes (HB-201) was evaluated in mice and resulted in a dose-dependent E7-specific CD8^+^ T cell response (39). A second administration of HB-201 further augmented antigen-specific CD8^+^ T cell responses, particularly in mice receiving high doses of HB-201.

The arenaviral vector system can induce T cells that produce multiple cytokines, which are referred to as polyfunctional. Polyfunctional T cells are not exhausted and provide a more robust, targeted immune response than those that produce a single cytokine. Treatment with HB-201 and HB-202, a PICV vector expressing the same HPV antigen as HB-201, resulted in the production of diverse pools of polyfunctional E7- and E6-specific CD8^+^ T cells as measured by their ability to produce varying levels of multiple cytokines, including tumor necrosis factor-α, interferon-γ, and interleukin (IL)-2 (4, 5, 39). An important discriminating feature of replication-competent arenavirus vectors is their ability to access specific lymphoid tissue microcompartments and infect resident fibroblastic reticular cells, which triggers the release of IL-33, a damage-associated molecular pattern or alarmin (**Figure 1B**). Previous studies have shown that IL-33 binds to ST2 receptors (IL1RL1, IL-33 receptor) on activated CTLs and enhances T cell expansion, differentiation, polyfunctionality, and antitumor efficacy (4, 5, 60). Immunization of mice with replication-competent HPV16 E7/E6-expressing LCMV and PICV vectors substantially augments CTL responses by triggering the IL-33/ST2 pathway (4, 60). Data with other replication-competent LCMV vectors, including an LCMV vector expressing ovalbumin, support these findings with respect to the induction of a polyfunctional effector CD8+ T cell pool and release of IL-33. Overall, these results demonstrate that arenavirus-mediated DC and T cell interactions, in combination with the presence of IL-33 and other pro-inflammatory cytokines, lead to a marked increase in antigen-specific, polyfunctional CD8^+^ T cells with antitumor activity. Antigen delivery systems that do not trigger this mechanism of immune activation (e.g., recombinant adenovirus- or poxvirus-based vectors) may not be able to activate a robust alarmin response, which may also limit size and polyfunctionality of the activated T cell pool (5).

As discussed previously, anti-vector immunity can inhibit viral delivery systems, impeding their readministration to augment immune responses. For other viral vector systems such as adenovirus-based vectors, the presence of preexisting adenovirus serotype 5 (Ad5) nAbs significantly impairs responses to recombinant Ad5-based vaccines (61). This led to the development of adenovirus vectors of non-human source, such as simian Ad backbones, which resulted in increased immunologic response rates in clinical trials (62, 63). However, vaccinations with simian Ad vectors also induce significant levels of vector nAbs and have therefore failed to demonstrate efficient boosting capacity of T cells after consecutive administrations (63–65).

Although arenaviruses typically do not induce significant vector nAbs in mice, anti-vector CTL responses were found to curtail immunogenicity when the same arenavirus vectors were administered for prime and boost injections. On repeat administration, vector backbone–specific responses often dominate over responses to the encoded transgene. This is especially true for tumor self-antigens. In contrast, administering an alternating sequence of two vectorized arenaviruses of distant genealogical relationship (such as a combination of Old World LCMV vectors and New World PICV vectors) impedes efficient boosting of vector backbone–directed CTL responses and focuses immune responses on the common transgene cargo. Applying this strategy in mice, alternating vector therapy using a LCMV vector (HB-201) and a PICV vector (HB-202) induced HPV16 E7/E6-specific CD8^+^ T cell responses that accounted for up to 50% of circulating CD8^+^ T cells, with similar levels observed when targeting tumor self-antigens such as P1A (4).

### Antigen-Specific Tumor Cell Killing

After activation and proliferation in lymphoid tissue, antigen-specific CTLs home to the site of the tumor and kill tumor cells presenting target antigens *via* MHC class I (**Figure 1C****)**. Treatment of mice with HB-201 not only induced high numbers of antigen-specific CD8^+^ T cells but also resulted in a significant accumulation of HPV16 E7-specific CD8^+^ T cells in the tumor tissues compared with the control group (**Figure 1C**) (39). Analogous results have been reported for mice carrying thymoma and mastocytoma tumors and in a mouse model of melanoma, in which arenavirus vectors expressing a cancer non–self-antigen (OVA), a cancer-testis self-antigen (P1A) or a differentiation self-antigen (Trp-2) significantly increased the number of TILs in tumors (4, 5, 66).

The antitumor activity of vector-encoded non–self-antigens was tested using HB-201 in the TC-1 mouse model of HPV+ cancers (**Figure 1C**). Compared with control-treated mice, treatment with HB-201 significantly suppressed tumor growth at all dose levels tested (39). Increasing doses of HB-201 significantly suppressed tumor growth in a dose-dependent manner that plateaued from 10^4^ to 10^6^ infectious vector units. Treatment with HB-201 also significantly prolonged survival in all mice treated with doses ≥10^4^ infectious vector units. The median survival time of vaccinated mice was >59 days (exceeding the duration of the study) versus 24 days for mice in the control group (39). Preclinical experiments using this HPV16 E7/E6-expressing vector showed direct links between immunogenicity, including increased levels of antigen-specific T cells and TILs, and antitumor activity (39).

### Memory T Cell Production and Persistent Immunity

Data from various preclinical experiments suggest that treatment with arenavirus vectors that express tumor antigens result in sustained CTL responses and durable antitumor activity indicative of immunologic memory (**Figure 1D**) (4, 5, 39). Specifically, HB-201 as well as alternating HB-202 and HB-201 treatment results not only in long-term presence of E7-specific CD8^+^ T cells but also in persistent antitumor activity. This suggests that the immunologic memory induced is clinically effective. Mice that were treated with HB-201 and cleared implanted tumors were rechallenged with the same tumor cells up to 198 days after the first tumor engraftment. These mice remained tumor free throughout the 150- or 210-day follow-up period, which indicates that tumor-specific T cells had formed a memory pool that resulted in effective rejection of a newly implanted tumor (39). Similar results were obtained in mice treated with an alternating regimen of PICV and LCMV vectors encoding a tumor self-antigen (4). Notably, the levels of memory T cells present after replication-competent arenavirus vector therapy increased with repeated doses of HB-201 and increased to even higher levels after an alternating sequence of HB-202 followed by HB-201 (4).

### Combination Immunotherapy Potential

Given the increasing role of immunotherapy, particularly programmed cell death 1 protein (PD-1) inhibitors, in the treatment of numerous advanced cancers, the potential of these replication-competent arenavirus-based immunotherapies in combination with a PD-1 inhibitor was also evaluated. In the TC-1 mouse model, an increase was observed in tumor clearance from 40% (4/10) with HB-201 alone to 78% (7/9) with HB-201 plus PD-1 inhibitor, suggesting that this combination has the potential to act synergistically and improve long-term outcomes. Treatment with a PD-1 inhibitor alone did not suppress tumor growth in this model, suggesting that the antitumor activity of HB-201 is enhanced by these agents (39). In addition, treatment of mice with either HB-201 alone or in combination with a PD-1 inhibitor resulted in a significant increase in HPV16 E7-specific CD8^+^ T cells in the tumor tissue (39). In addition to high numbers of tumor-specific T cells, these T cells must maintain their functionality in the immunosuppressive milieu of the TME to overcome tumor resistance mechanisms and to efficiently kill cancer cells. Therefore, additional combinations of arenavirus vectors and immune checkpoint inhibitors are currently under evaluation in preclinical models. A portion of the ongoing phase 1/2 study, described in the Discussion section, will evaluate the efficacy of HB-201/HB-202 alone and in combination with a PD-1 inhibitor to determine if these preclinical data translate to the clinic (3).

## Discussion

Overall, in several preclinical cancer models, arenavirus vectors expressing viral- and self-antigens have induced potent antigen-specific CD8^+^ T cell responses and antitumor activity. Preclinical data have demonstrated a substantial increase in antigen-specific TILs in response to therapy with replication-competent arenavirus vectors expressing several different tumor antigens (5, 39). Taken together, a range of mechanistic preclinical studies have documented the immunogenicity and antitumor activity of arenavirus vectors that express a broad range of tumor antigens. Specifically, we have shown that engineered replication-competent arenavirus vectors target APCs to deliver encoded tumor antigens, induce potent antigen-specific CD8^+^ T cell responses, and enhance T cell infiltration into tumors. We have demonstrated that these properties can be augmented by repeat injections and maintained long term (4, 39). Based on these preclinical results, this arenavirus platform has the potential to become a foundation for the development of therapies that encode various types of cancer antigens and can target a broad range of tumor types.

### Future Directions and Clinical Development

Several investigational products are currently being developed using engineered replication-competent arenavirus, including the clinical stage vectors HB-202/HB-201 for HPV+ cancers and vectors that express key prostate cancer antigens (prostate-specific antigen, prostatic acid phosphatase, and prostate-specific membrane antigen) that are nearing the clinic for the treatment of prostate cancer.

HPV infections, particularly infections with HPV16, are a major cause of several human cancers including head and neck squamous cell carcinoma (HNSCC), cervical cancer, and anogenital cancers (67, 68). Despite the availability of preventive HPV vaccines, HPV prevalence remains high and HPV-associated cancers remain a significant health concern worldwide (67, 68). While numerous treatments are currently approved for use in HPV-associated cancers, including epidermal growth factor receptor inhibitors and immunotherapies, high rates of recurrence and disease progression require additional therapeutic options (69).

**Figure 2:**
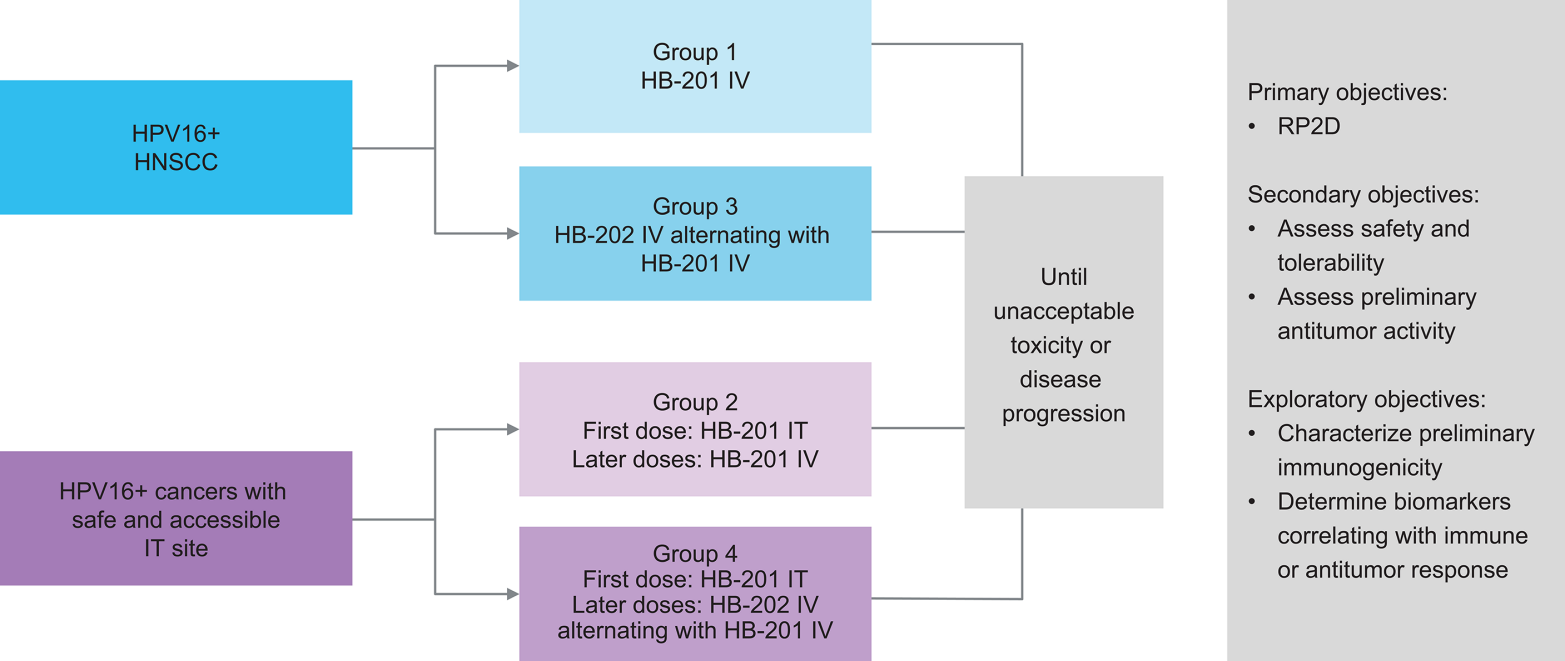
Design of ongoing phase 1/2 study of HB-201 in patients with previously HPV+ cancers, including HNSCC (3, 42). ^a^ 3 + 3 dose escalation with additional biomarker- and schedule-finding cohorts. ^b^ All injections consist of specified vector monotherapy. HNSCC, head and neck squamous cell carcinoma; HPV, human papilloma virus; IT, intratumoral; IV, intravenous; q2w, every 2 weeks; q6w, every 6 weeks; RP2D recommended phase 2 dose.

Given the promising preclinical data and the high unmet need for novel treatments for HPV+ cancers, a phase 1/2 study was initiated with the HPV16 E7/E6 fusion protein–expressing vectors HB-201 (LCMV) and HB-202 (PICV). This study (NCT04180215), which began in 2019, is exploring treatment with HB-201, alternating sequences of HB-202 and HB-201, and combinations with a PD-1 inhibitor in patients with previously treated advanced or metastatic HPV16+ HNSCC or other cancers (**Figure 2**) (3, 42). Patients eligible for this study are required to have received at least one line of prior standard therapy (e.g., platinum-based chemotherapy and/or anti–PD-1 therapy). The phase 1 portion of this study is evaluating escalating doses of HB-201 monotherapy and alternating doses of HB-202 and HB-201. This part of the study is assessing two different modes of administration. Some cohorts will receive all doses IV while others will receive an initial dose intratumorally in needle-accessible tumors, followed by IV doses. The primary objective of the phase 1 portion of the study is to identify the recommended phase 2 dose based on clinical responses and potential dose-limiting toxicities. The primary objective of the phase 2 expansion phase is to assess preliminary antitumor activity based on objective response and disease control rates. Secondary endpoints include safety and tolerability and antitumor activity as measured by objective response and disease control rates. duration of response, and progression-free and overall survival.

As of March 31, 2021, 38 patients (84% with HNSCC) have been enrolled in the phase 1 portion of the study and have been treated with HB-201 monotherapy or HB-202 and HB-201 alternating therapy. Among the patients with HNSCC enrolled, the median number of prior therapeutic regimens was three and approximately 90% of patients had previously received a checkpoint inhibitor and/or prior platinum. Preliminary but promising safety, efficacy, and immunogenicity data were presented at the American Association for Cancer Research and American Society of Clinical Oncology annual meetings in 2021 (41, 42). On day 4 after a single IV injection of HB-201, nearly all patients tested had an increase in key systemic pro-inflammatory cytokines and chemokines. An assessment of peripheral blood mononuclear cells by direct interferon-γ ELISpot showed a strong HPV16 E7/E6-specific CD8^+^ T cell response that was detected on day 15 after a single dose of HB-201 or HB-202. In the available data, the highest increase seen in antigen-specific CD8+ T cells with HB-201 was from 20 to 3164 spot forming units per 10^6^ cells at baseline and day 15, respectively; for HB-202, spot forming units per 10^6^ cells increased from 0 at baseline to >6000 at day 15. Assessment of intracellular cytokine staining showed that circulating E7/E6-specific CD8^+^ T cells expressing interferon-γ and/or tumor necrosis factor-α had increased up to 4% and 8% with HB-201 and HB-202 monotherapy, respectively, and was further elevated up to 40% with HB-202 and HB-201 alternating therapy (41, 42). These results showing robust antigen-specific CD8^+^ T cell response in patients with advanced disease is a distinguishing feature of this arenavirus vector technology, a result which has yet to be demonstrated by other antigen delivery systems. Among the 11 evaluable patients with HPV+ HNSCC who received the most promising regimen of HB-201 based on these preliminary data (all IV injections administered every 3 weeks), the overall response rate was 18% with 2 patients having partial responses, the disease control rate was 73%, and median progression-free survival was 3.45 months. These responses are equivalent or superior to those reported with checkpoint inhibitors in recurrent and metastatic HPV+ HNSCC but in earlier lines of treatment (70–72). The alternating HB-202/HB-201 cohort is actively enrolling patients. However, at the time of this data cutoff, these patients had generally not yet been followed long enough to assess efficacy. In addition to the promising efficacy results seen to date with single vector therapy, the arenavirus vectors used in this study had an acceptable safety profile with no treatment-related dose-limiting toxicities, grade ≥3 adverse events, serious adverse events, or discontinuations due to adverse events (41). Recruitment for this study is ongoing, and additional immunogenicity and efficacy data from other treatment cohorts are eagerly anticipated.

### Conclusion

Engineered arenavirus-based vectors are emerging as a very promising systemically administered, non-lytic viral immunotherapeutic strategy for the treatment of cancer. This arenavirus vector platform can be engineered to express a broad range of antigens. To date, replicating arenavirus vectors expressing self- and non–self-antigens has been shown to trigger an exceptionally strong activation of antigen-specific CD8^+^ T cells that localize to the TME and induce a durable antitumor response. Early-stage clinical results of this technology for the treatment of previously treated and clinically advanced HPV16+ cancers demonstrate robust immunogenicity and promising clinical efficacy and safety supporting the translation of preclinical mechanisms to human therapeutic activity. These data are not only exciting for patients with advanced HPV+ tumors, but they also provide a strong clinical proof-of-concept for broad expansion of this foundational technology platform into other tumor types using various tumor antigens.

## Author Contributions

HL, IM, DP, and KKO contributed to conception and wrote sections of the manuscript. All authors were involved in the content, writing, and revising of this review article. All authors contributed to the article and approved the submitted version.

## Funding

This work was funded by Hookipa Pharma Inc.

## Conflict of Interest

Author HL, SS, KK, XQ, AH, KS, UB, JR, SA-E, TS, FS, IM and KKO are employees of Hookipa and own stock. CI is an employee of Hookipa who received travel funds and owns stock. DP owns stock in Hookipa and received funding from Hookipa for scientific consultancy, travel funds, writing support, and patent licensing fees.

The authors declare that this study received funding from Hookipa Pharma Inc. The funder had the following involvement with the study: study design, collection, analysis, interpretation of data, the writing of this article and the decision to submit it for publication.

## Publisher’s Note

All claims expressed in this article are solely those of the authors and do not necessarily represent those of their affiliated organizations, or those of the publisher, the editors and the reviewers. Any product that may be evaluated in this article, or claim that may be made by its manufacturer, is not guaranteed or endorsed by the publisher.
